# Direct characterization of photoinduced lattice dynamics in BaFe_2_As_2_

**DOI:** 10.1038/ncomms8377

**Published:** 2015-06-08

**Authors:** S. Gerber, K. W. Kim, Y. Zhang, D. Zhu, N. Plonka, M. Yi, G. L. Dakovski, D. Leuenberger, P.S. Kirchmann, R. G. Moore, M. Chollet, J. M. Glownia, Y. Feng, J.-S. Lee, A. Mehta, A. F. Kemper, T. Wolf, Y.-D. Chuang, Z. Hussain, C.-C. Kao, B. Moritz, Z.-X. Shen, T. P. Devereaux, W.-S. Lee

**Affiliations:** 1 Stanford Institute for Materials and Energy Sciences, SLAC National Accelerator Laboratory and Stanford University, 2575 Sand Hill Road, Menlo Park, California 94025, USA; 2 Department of Physics, Chungbuk National University, 52 Naesudong-ro, Heungdeok-gu, Cheongju 361-763, Korea; 3 Advanced Light Source, Lawrence Berkeley National Laboratory, 1 Cyclotron Road, Berkeley, California 94720, USA; 4 Linac Coherent Light Source, SLAC National Accelerator Laboratory, 2575 Sand Hill Road, Menlo Park, California 94025, USA; 5 Departments of Physics and Applied Physics, Stanford University, 476 Lomita Mall, Stanford, California 94305, USA; 6 Stanford Synchrotron Radiation Lightsource, SLAC National Accelerator Laboratory, 2575 Sand Hill Road, Menlo Park, California 94025, USA; 7 Computational Research Division, Lawrence Berkeley National Laboratory, 1 Cyclotron Road, Berkeley, California 94720, USA; 8 Institute for Solid State Physics, Karlsruhe Institute of Technology, Hermann-v.-Helmholtz-Platz 1, 76021 Karlsruhe, Germany; 9 SLAC National Accelerator Laboratory, 2575 Sand Hill Road, Menlo Park, California 94025, USA; 10 These authors contributed equally to this work

## Abstract

Ultrafast light pulses can modify electronic properties of quantum materials by perturbing the underlying, intertwined degrees of freedom. In particular, iron-based superconductors exhibit a strong coupling among electronic nematic fluctuations, spins and the lattice, serving as a playground for ultrafast manipulation. Here we use time-resolved X-ray scattering to measure the lattice dynamics of photoexcited BaFe_2_As_2_. On optical excitation, no signature of an ultrafast change of the crystal symmetry is observed, but the lattice oscillates rapidly in time due to the coherent excitation of an *A*_1*g*_ mode that modulates the Fe–As–Fe bond angle. We directly quantify the coherent lattice dynamics and show that even a small photoinduced lattice distortion can induce notable changes in the electronic and magnetic properties. Our analysis implies that transient structural modification can be an effective tool for manipulating the electronic properties of multi-orbital systems, where electronic instabilities are sensitive to the orbital character of bands.

## Abstract (web summary)

In BaFe_2_As_2_, the lattice couples strongly to the magnetic and electronic degrees of freedom, providing a way to control them. Here, by means of time-resolved X-ray scattering, the authors measure rapid lattice oscillations, which can induce changes in the material's electronic and magnetic properties.

## Graphical abstract

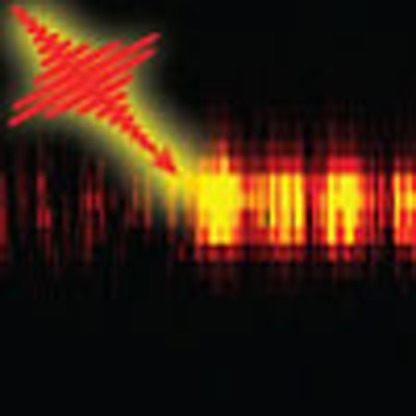

One of the goals in materials research is to control quantum phases that emerge in strongly correlated materials, such as superconductivity and magnetism, since many of them exhibit exotic properties that promise applications in technology^1^. While the microscopic mechanism of such emergence remains elusive, it is generally agreed that the formation and competition of quantum phases results from a subtle balance among the strongly coupled spin, charge, lattice and orbital degrees of freedom. Shifting this balance provides a promising avenue to manipulate emergent phenomena in strongly correlated materials.

In equilibrium the electronic properties are typically modified by chemical doping or application of an external parameter, for example, magnetic fields, strain or hydrostatic pressure^2^,^3^,^4^. However, perturbing the subtle balance of interactions by using ultrafast light pulses to manipulate material properties in non-equilibrium transient states has recently received significant attention. Many studies, including the generation of coherent collective oscillatory states^5^,^6^,^7^,^8^ and transiently induced phases which have no analogue in thermal equilibrium^9^,^10^,^11^, have demonstrated the power of these techniques. To date, most information about these photoinduced states is obtained by optical or photoemission spectroscopy, providing only limited and indirect insight on the dynamics of the lattice degree of freedom. Therefore, it is important to directly probe the complementary structural dynamics of these photoinduced states via time-resolved X-ray scattering with femtosecond resolution.

BaFe_2_As_2_, a parent compound of the high-temperature superconducting iron pnictides^4^,^12^, is an ideal system for manipulating electronic properties via transient structural modification, as the lattice couples strongly to the magnetic and electronic degrees of freedom. On cooling, the system first undergoes a structural phase transition (*T*_s_), followed by a spin-density wave (SDW) transition^13^,^14^ at *T*_N_, just 0.75 K below *T*_s_. Importantly, the existence of electronic nematic fluctuations has been demonstrated at even higher temperatures^15^,^16^,^17^,^18^; and the divergence of the nematic susceptibility drives the aforementioned structural phase transition. The electronic structure of pnictides also appears to be extremely sensitive to the Fe–As–Fe bond angle *α* (Fig. 1a) as it changes the hybridization of the iron 3*d* and arsenic 4*p* orbitals—evidenced by band structure calculations^19^,^20^—and correlates with the superconducting transition temperature in doped compounds^21^,^22^ as well as magnetism^23^. Notably, transient optical reflectivity^24^, conductivity^11^ and time- and angle-resolved photoemission spectroscopy^25^,^26^ (trARPES) revealed that an ultrafast optical excitation induces coherent oscillations with a frequency *f*=5.45 THz, corresponding to an *A*_1*g*_ phonon mode observed in Raman spectroscopy^27^. Intriguingly, THz spectroscopy^11^ indicates that exciting the coherent *A*_1*g*_ phonon mode enhances magnetism by inducing a transient SDW state even above *T*_N_; and trARPES^25^,^26^ finds concomitant strong modulations of the density of states near the Fermi level for similar excitations. However, disentangling the lattice's influence requires a direct structural characterization in the photoinduced transient state, which serves as an important experimental boundary condition for the associated variation of the electronic and magnetic degrees of freedom.

We employ time-resolved X-ray scattering at the Linac Coherent Light Source (LCLS), an X-ray free-electron laser (FEL), to directly measure the photoexcited lattice dynamics in BaFe_2_As_2_. We map the temporal evolution of the crystal structure by recording the diffraction pattern at different time delays Δ*t* between an 800 nm infrared (IR) pump pulse and the 8.7 keV X-ray probe pulse (Fig. 1b). Recently, Rettig *et al*.^28^ conducted a similar study to extract the change of *α* due to coherent excitation of the *A*_1*g*_ phonon. There the role of the *A*_1*g*_ mode on the electron–phonon coupling is discussed. Photoinduced SDW order is only commented based on empirical rules deduced from doped compounds. Here we corroborate these findings, while also illuminating different experimental aspects and elucidating the impact of the lattice dynamics on the electronic and magnetic properties. In particular, we investigate two questions: Can the ultrafast photoexcitation trigger an ultrafast change of the crystal symmetry by perturbing the electronic nematic state? How are the 5.45 THz coherent oscillations, as seen in both optical and photoemission spectroscopy, reflected in the lattice degree of freedom, and what are the consequences on the electronic and magnetic properties? We present a microscopic model which incorporates the transient lattice distortion deduced from our measurements and calculate the change of the band structure, Fermi surface nesting properties and the SDW transition temperature. Our analysis suggests new opportunities of manipulating the properties of multi-orbital systems via photoinduced transient lattice modification.

## Results

### Photoinduced lattice dynamics below *T*_s_

Figure 2 shows the temperature dependence of the (118)_T_ lattice Bragg peak (in tetragonal notation) near the structural (*T*_s_) and the antiferromagnetic phase transition (*T*_N_) during slow cooling from a nominal temperature of *T*=140 to 137 K. For *T*>*T*_s_ the crystal structure is tetragonal, yielding a single peak on the detector (Fig. 2a). On cooling, the (118)_T_ peak splits (Fig. 2b,c), as a consequence of the tetragonal to orthorhombic structural phase transition (space group: *I*4/*mmm* → *Fmmm*). The detailed evolution of the peak-splitting near *T*_s_ and *T*_N_ is depicted in Fig. 2d: it occurs continuously for temperatures *T*_s_>*T*>*T*_N_, followed by a sudden jump at the SDW ordering temperature *T*_N_. This behaviour is equivalent to the results obtained in thermal equilibrium using a synchrotron X-ray source^14^.

On photoexcitation via femtosecond optical pulses, hot electrons are generated, populating states above the Fermi level, which then decay through allowed electron–electron and electron–phonon scattering channels. These incoherent scattering processes should, in principle, weaken the nematic fluctuations, which may allow the crystal structure to recover the original fourfold symmetry, that is, the tetragonal phase.

To test the aforementioned conjecture, Fig. 3a,b shows the temporal evolution of the split (118)_T_ peaks for a temperature *T*_s_>*T*>*T*_N_, along a line cut on the area detector (indicated in the inset). As a function of time the two orthorhombic peaks neither merge nor come closer, showing no signature of any ultrafast structural change from orthorhombic to tetragonal symmetry within Δ*t*=4.5 ps after photoexcitation. Also, no evidence is found for a change of the lattice parameters in the picosecond regime, as the profile of the Bragg peaks does not shift nor broaden (Fig. 2b). Therefore, we conclude that a structural transition, a process that involves the movement of all atoms to eliminate the orthorhombic structural domains^29^ and also depends on the strain potential from the bulk material, does not occur in BaFe_2_As_2_ on these ultrafast time scales at an absorbed fluence of 2.9 mJ cm^−2^—approximately half of the sample damage threshold observed in the experiment.

### Direct quantification of the coherent lattice dynamics

Careful examination of the diffraction pattern as a function of time reveals ultrafast lattice dynamics. As shown in Fig. 3c, the intensity of both split Bragg peaks exhibits a time-dependent modulation following photoexcitation, suggesting that the entire probed sample volume is in a coherent oscillatory state with a period of ∼185 fs.

This coherent state is characterized further at a slightly elevated temperature *T*>*T*_s_, where the improved signal-to-noise ratio facilitates a quantitative analysis, as the scattered intensity is concentrated in one single Bragg peak. Figure 4 shows the temporal evolution of the (118)_T_ line cut (Fig. 4a) and the integrated counts on the area detector (Fig. 4b), normalized to the intensity before time zero. Most striking is the rise of the (118)_T_ Bragg peak intensity with a maximum at Δ*t*∼130 fs after photoexcitation. Moreover, coherent oscillations are resolved with a periodicity of 185 fs, as already evidenced in Fig. 3c. The Fourier transform (inset of Fig. 4b) and the background-subtracted diffracted intensity (Fig. 4c) yield an oscillation with *f*=5.45(4) THz that coincides with the frequency of the *A*_1*g*_ phonon mode, as measured by Raman spectroscopy^27^. This finding provides strong support that the coherent oscillations indeed can be attributed to the Fe–As–Fe bond angle mode. We note that the generation of coherent *A*_1*g*_ phonons is consistent with the ‘displacive excitation' mechanism described by Zeiger *et al*.^30^. The decay of the (118)_T_ background is caused by the increase of the Debye–Waller effect due to incoherent phonon proliferation via hot electron scattering.

**Figure 1:**
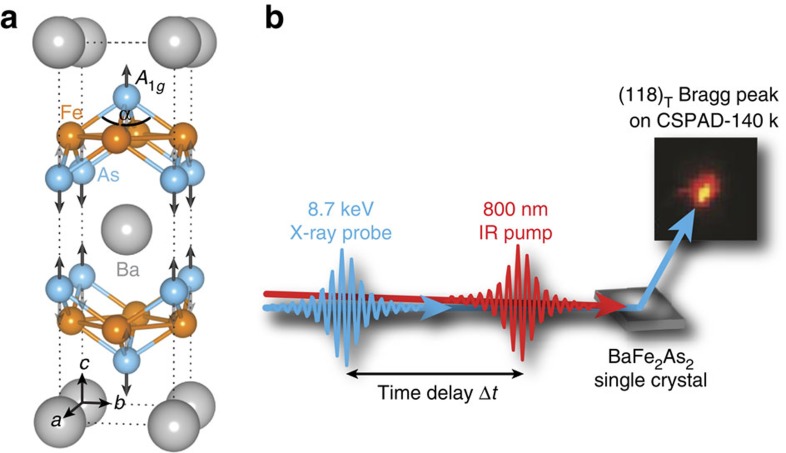
Crystal structure and time-resolved X-ray scattering. (**a**) Tetragonal crystal structure of BaFe_2_As_2_ in the presence of the *A*_1*g*_ phonon mode, parametrized by the Fe–As–Fe bond angle *α*. (**b**) Schematic of the experimental setup with the incoming infrared (IR) pump (red) and the X-ray probe pulse (blue). The temporal evolution of the diffraction pattern from the photo-excited BaFe_2_As_2_ single crystal was measured with a CSPAD-140k area detector. Δ*t* is the time delay of the probe pulse with respect to the pump pulse.

**Figure 2:**
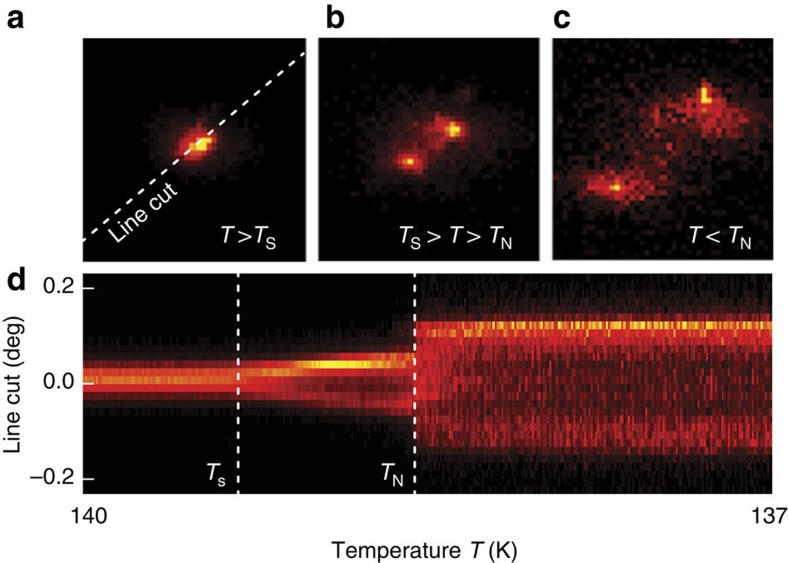
Structural phase transitions without optical pumping. (**a**–**c**) Diffraction pattern of the (118)_T_ lattice Bragg peak at temperatures in the vicinity of the structural (*T*_s_) and magnetic (*T*_N_) phase transition. (**d**) Line cut on the area detector (dashed line in **a**) slowly cooling from a nominal temperature of *T*=140 to 137 K. The tetragonal (118)_T_ Bragg peak splits first at *T*_s_, due to the transition to the orthorhombic crystal structure, and then further at *T*_N_ as a result of the onset of SDW order.

**Figure 3:**
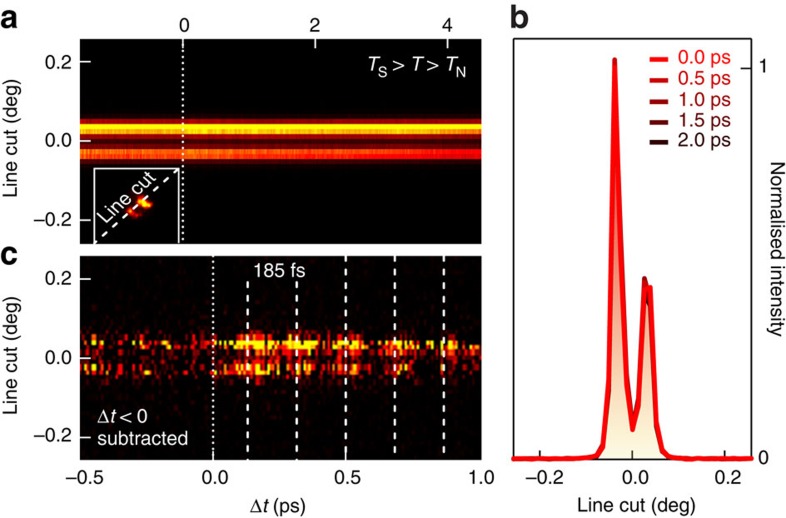
Photo-excited lattice dynamics below ***T***_**s**_. (**a**) Temporal evolution of the line cut through the split Bragg peak at *T*_s_>*T*>*T*_N_ at an absorbed pump fluence of 2.9 mJ cm^−2^. The inset depicts the line cut on the area detector. (**b**) Diffraction peak profiles along the line cut at selected delay times. No changes are observed in peak position and width. (**c**) Subtraction of the averaged line cuts before time zero (Δ*t*=−0.5 to 0 ps) reveals a photo-excited periodic intensity modulation of both orthorhombic domains for positive time delays.

To better understand and quantify the lattice dynamics associated with the coherent excitation of the *A*_1*g*_ phonon, we have performed a structure factor calculation. Since the associated eigenmode involves only the vertical displacement of the arsenic atoms, the structural change can be parametrized by the Fe–As–Fe bond angle *α* (Fig. 1a). In the presence of the *A*_1*g*_ bond angle mode the structure factor can be written as


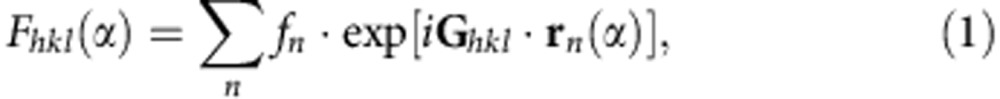


where *n* indexes individual atoms in the unit cell, *f*_*n*_ is the dispersion-corrected atomic scattering factor^31^, **r**_*n*_(*α*) is the atomic position and **G**_*hkl*_ is the scattering vector. The *α*-dependent diffracted intensity is obtained from the relation 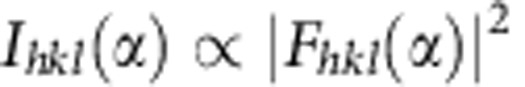.

**Figure 4:**
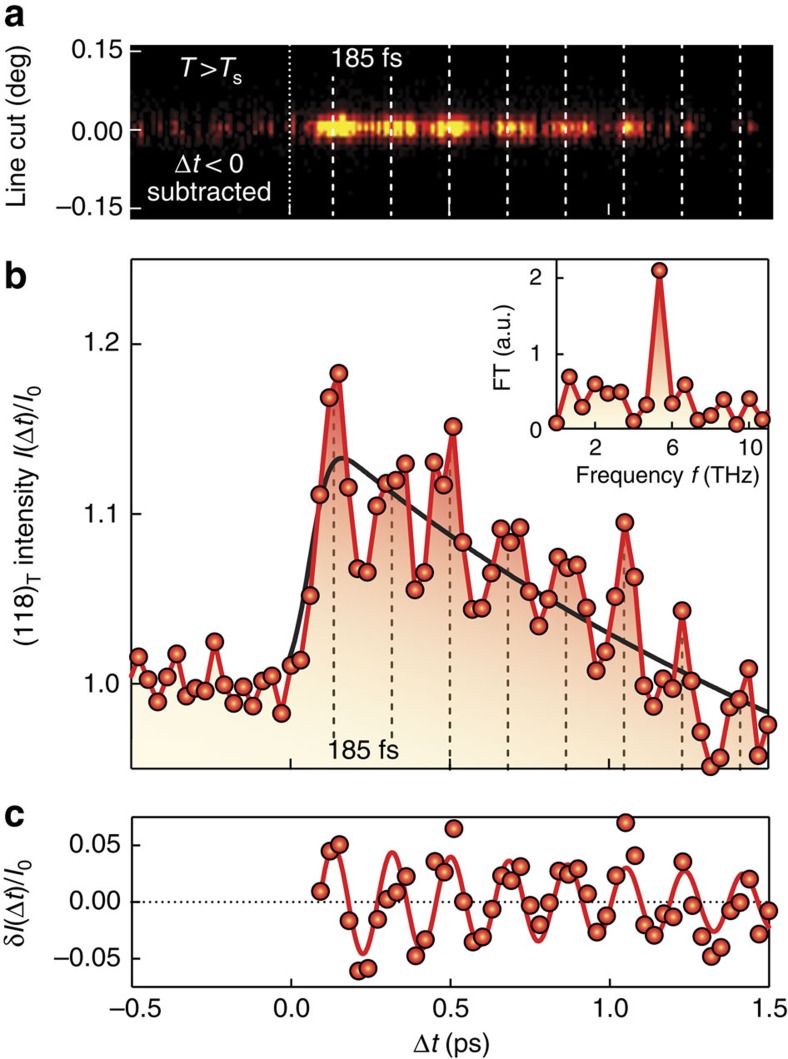
Photo-excited coherent lattice dynamics for ***T*****>*****T***_**s**_. (**a**) Temporal evolution of the line cut after subtraction of the averaged line cuts before time zero. (**b**) Integrated intensity on the area detector as a function of time. Both **a** and **b** show a distinct modulation of the (118)_T_ Bragg peak intensity after photoexcitation. Time zero (Δ*t*=0) is defined as the time delay, at which one observes a rise of the diffracted Bragg peak intensity. The background (black line) is modelled as a convolution of the overall time resolution and an exponential decay of the initial ultrafast intensity rise, on a linear slope. (**c**) The background-subtracted integrated intensity and the Fourier transform (FT, inset of **b**) both identify the coherent oscillations with the 5.45(4) THz *A*_1*g*_ phonon mode. The frequency error from a damped oscillator fit to the background-subtracted integrated intensity *δI*(Δ*t*)/*I*_0_ (red line in **c**) corresponds to 1 s.d.

**Figure 5:**
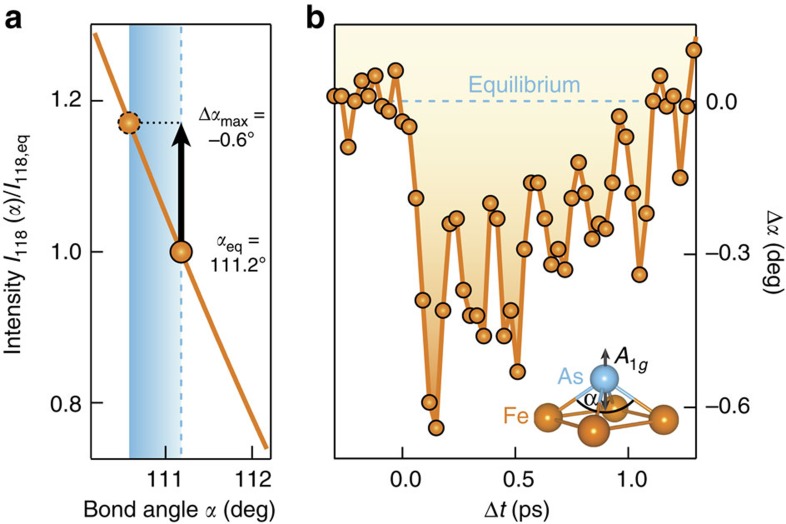
Dynamics of the Fe–As–Fe bond angle. (**a**) Dependence of the (118)_T_ Bragg peak intensity on the bond angle *α* from a structure factor calculation (orange line). The shaded area indicates the magnitude of the initial change Δ*α*_max_=−0.62(4)°, as obtained by comparison with the maximal intensity change of the integrated Bragg peak intensity in Fig. 4b. (**b**) The deduced temporal evolution Δ*α*(*t*) from the raw data (without deconvolution of the finite time resolution), reveals an *A*_1*g*_ oscillation amplitude Δ*α*_osc_=0.27(8)°, following the initial decrease of *α*. The errors of Δ*α*_max_ and Δ*α*_osc_ correspond to 1 s.d.

The calculated relative intensity change 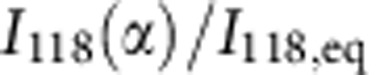 is shown in Fig. 5a. The signal clearly increases from its equilibrium value with decreasing *α*. A comparison with the raw data in Fig. 4b reveals that the initial ultrafast increase of the (118)_T_ Bragg peak intensity is associated with an ultrafast decrease of the bond angle Δ*α*_max_=−0.62(4)°. Figure 5b depicts the temporal evolution of the bond angle change Δ*α*(*t*), as deduced from the raw data shown in Fig. 4b without deconvolution of the finite time resolution, revealing an *A*_1*g*_ oscillation amplitude Δ*α*_osc_=0.27(8)° (averaged amplitude of the first three oscillations), in addition to the initial decrease of *α*. The magnitude of Δ*α*_osc_ is in agreement with the results obtained by Rettig *et al*.^28^, after taking into account the pump fluence and the time resolution of the probe pulse. For clarity, we note that in ref. 28 the *A*_1*g*_ mode is parametrized in terms of the Fe–As tetrahedral angle and not the Fe–As–Fe bond angle. The experimentally established temporal dependence of the bond angle provides direct input for a theoretical evaluation of the associated transient variation of the electronic and magnetic degrees of freedom in this coherent oscillatory state.

### Consequence on the electronic and magnetic properties

**Figure 6:**
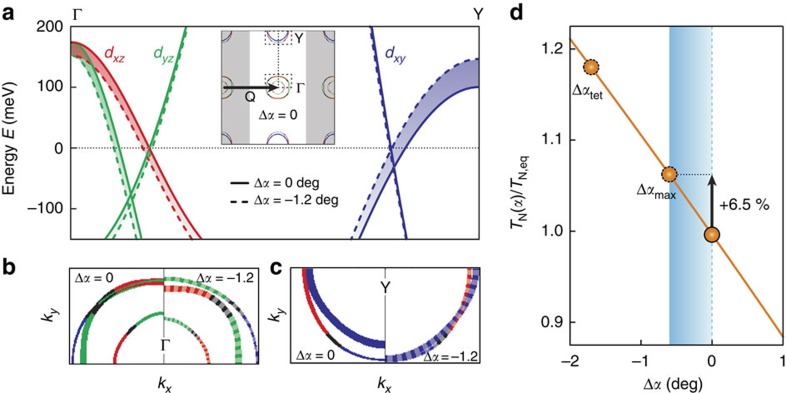
Influence of *α* on the electronic structure and SDW order. (**a**) Effect of Δ*α*=−1.2° on the low-energy bands along the Γ–Y momentum cut in the SDW-folded Brillouin zone. The equilibrium bands (solid lines) shift as a result of the change in the Fe–As–Fe bond angle (dashed lines). The dominant (>50%) *d*-orbital character for each band is colour coded, that is, *d*_*xz*_ (red), *d*_*yz*_ (green) and *d*_*xy*_ (blue). We choose twice the experimentally observed Δ*α*_max_ to better illustrate the qualitative change. The inset shows the equilibrium Fermi surface and the locations of the Γ- and Y-points. The unshaded area represents the **Q**=(*π*, 0) SDW-folded Brillouin zone. (**b**,**c**) Enlarged portions of the squares (dashed lines) in **a** show the effect of Δ*α*=−1.2° on the Fermi surface in the SDW-folded Brillouin zone. Equilibrium Fermi surface pockets (left half of each panel) shift to new positions under the change of *α* (right half of each panel). Improved nesting of bands with similar orbital character (*d*_*xy*_) is observed at the Y-point. (**d**) Results of self-consistent Hartree–Fock mean-field calculations for the relative change of *T*_N_ as a function of Δ*α*. The arrow indicates a 6.5% increase in *T*_N_ for the experimentally observed Δ*α*_max_≈−0.6°. *α*_tet_ is the Fe–As–Fe bond angle for a regular FeAs_4_ tetrahedron, where superconductivity is found to be maximal in iron-based compounds^21^,^22^. The orange line is a fit through the full data set as obtained from the mean-field calculations.

To assess the qualitative influence of the transient modification of the crystal structure on magnetism^11^, we have carried out self-consistent Hartree–Fock mean-field calculations. We employ a five-orbital, tight-binding fit to the density functional theory-derived band structure^32^ of LaFeAsO, which shows a qualitative similarity to BaFe_2_As_2_ at low doping and for energies near the Fermi level^33^. This simplifies the discussion by restricting the calculations to two dimensions. Throughout the analysis we reference to the one iron Brillouin zone notation, which provides additional clarity when discussing the evolution of the band structure and Fermi surfaces as a function of *α*. The magnetic moment, and hence the Neel temperature *T*_N_, is determined at the mean-field level for a multi-orbital electron–electron interaction with parameters tuned to stabilize a **Q**=(*π*, 0) SDW with six electrons per site^34^,^35^. We assume that the pnictogen height, which controls the bond angle *α*, primarily affects the band structure parameters associated with the *d*_*xy*_ orbital: the nearest and next-nearest neighbour hopping integrals^19^,^36^. While the pnictogen height also affects other parameters, these changes are shown to have more influence at higher binding energies and less on the band structure close to the Fermi level^19^,^26^. To mimic the bond angle in BaFe_2_As_2_, we subtract 2.4° from the equilibrium value of *α* in LaFeAsO and extrapolate the intra-orbital *d*_*xy*_ hopping integrals linearly over a range of *α* following the dependence determined from ref. 19. We note that the derived band structure (Fig. 6a) qualitatively agrees with the known SDW-folded band structure^37^,^38^.

Figure 6a shows the influence of Δ*α*=−1.2°, on the band structure close to the Fermi level in the SDW (*π*, 0)-folded zone. A value of Δ*α*=−1.2° was used for clearer visualization of the photoinduced changes in the band structure and Fermi surface. Since the photoinduced effects essentially have a linear dependence with Δ*α*, the shaded areas are about half the size for Δ*α*=−0.6° (the experimental value). Principally, the change in *α* raises the dominant *d*_*xy*_ hole-band near the Y-point (folded from the one iron Brillouin zone M-point), consistent with the change in *d*_*xy*_ hopping integrals^19^. To maintain a consistent filling fraction, a rigid chemical potential shift leads to a lowering of the *d*_*xz*_ and *d*_*yz*_ bands at the Γ-point, which is consistent with trARPES^25^,^26^, and not connected to the doping evolution of the equilibrium state. The significance of these changes becomes apparent when viewed on the Fermi surface (inset of Fig. 6a). Figure 6b,c depicts the Fermi surface in the vicinity of the Γ- and Y-points in the SDW-folded Brillouin zone. A reduction of the Fe–As–Fe bond angle improves nesting at the Y-point considerably, where hole- and electron-bands of *d*_*xy*_ character interact. While the change reduces nesting at the Γ-point, this has less impact on magnetism due the incompatibility of orbital band characters, which already suppresses the opening of a SDW gap there. These effects are borne out by the change in the calculated Neel temperature as a function of *α*, shown in Fig. 6d.

## Discussion

In general, our results highlight that coherent excitation of an optical phonon may allow manipulation of the electronic properties of multi-orbital systems, in which orbital physics is central to the electronic structure. In such compounds, electronic instabilities are driven by band edges with different orbital character close to the Fermi level, which are sensitive to small changes of the underlying crystal structure. Remarkably, Fig. 6d shows that Δ*α*_max_=−0.62° as induced by photoexcitation (corresponding to a relative change of 0.6%), results in a substantial enhancement of ∼6.5% in the calculated SDW transition temperature (Δ*T*_N_∼9 K) precisely due to these effects. This is qualitatively consistent with the recent observation^11^ of photoinduced transient SDW order at temperatures above *T*_N_. Although the onset of SDW order and the change in crystal symmetry are coupled in equilibrium, such a coupling might not hold in the photoexcited non-equilibrium state which may depend on the nature of the transient SDW order, for example, fluctuating or static, and associated time scales. We note that the mean-field calculations do not include effects of fluctuations, which are crucial for short-range SDW correlations; and while the transient Fermi surface topology favours the emergence of SDW order, it does not take into account scattering processes due to the relaxation of photoexcited ‘hot' electrons.

Nevertheless, knowing the dramatic transient variation of the *d*_*xy*_ bands with Δ*α*, one can already envision some exciting possibilities. Lifshitz transitions have been identified^39^,^40^ in the temperature-doping phase diagram of Ba(Fe_1−*x*_Co_*x*_)_2_As_2_ compounds, associated with the doping-dependent hybridization between *d*_*xy*_ bands and others. It is conceivable that the coherent excitation of the *A*_1*g*_ phonon could drive a transient Lifshitz transition in compounds near these doping concentrations. Furthermore, the *d*_*xy*_ bands in the isostructural compound *A*_*x*_Fe_2−*y*_Se_2_ (*A*=K,Rb) are strongly renormalized due to correlation effects^41^. An ultrafast modulation of the Fe–Se–Fe bond angle may be employed as a novel tuning parameter to alter these correlation effects and modify the associated electronic properties. In addition, given the sizeable effect on the electronic and magnetic properties, it would be tantalizing to investigate how this transient coherent oscillatory state affects superconductivity. Theoretical and experimental studies already suggest an intimate connection between the Fe–As–Fe bond angle, the superconducting transition temperature^21^,^22^, and the symmetry of the superconducting order parameter^19^.

## Methods

### Experimental setup

The BaFe_2_As_2_ single crystal was grown from self-flux and was of a millimetre size. It had a plate-like shape with the tetragonal *c*-axis perpendicular to the scattering surface that was prepared by cleaving. The lattice dynamics of the photoexcited single crystal were studied at the X-ray Pump–Probe (XPP) instrument of the LCLS X-ray FEL^42^ at the SLAC National Accelerator Laboratory, benefiting from superb time resolution and X-ray pulse intensity. A dedicated sample chamber was assembled, allowing for low-temperature pump–probe hard X-ray scattering. All data reported here were measured at nominal temperatures *T*=137–140 K. Throughout the manuscript errors correspond to 1 s.d. The Python package periodictable 1.4.1 was used to compute the dispersion-corrected atomic scattering factors^31^ in the structure factor calculation.

### Pump–probe configuration

The BaFe_2_As_2_ single crystal was excited with an optical pump pulse and, thereafter, probed by a hard X-ray pulse. Both were operated with a repetition rate of 120 Hz. The pump laser provided a *p*-polarized 800 nm infrared pulse with a duration of ∼55 fs. The angle of incidence was 2° with a spot size of 65 × 80 μm^2^ (*h* × *υ*, Gaussian full-width at half-maximum), yielding an absorbed fluence of 2.9 mJ cm^−2^. As the probe, *p*-polarized *E*=8.7 keV X-rays from a silicon (111) monochromator, with a pulse duration of ∼45 fs, were used. A combination of upstream slits and beryllium compound refractive lenses shaped the X-ray beam to 25 × 50 μm^2^, to fit the photoexcited sample volume at 0.5° grazing incidence. 8.7 keV X-rays were used to match the penetration depths of the pump and probe pulses. The experimental geometry resulted in a flux of ∼10^8^ photons per pulse on the sample—well below the damage threshold. The arrival time between the pump laser and the X-rays was measured pulse by pulse to allow for time sorting^43^ that mitigates the intrinsic jitter of the FEL and yields an overall time resolution of better than 75 fs. The X-ray diffraction patterns were recorded using a CSPAD-140k detector^44^ at full beam rate.

## Additional information

**How to cite this article:** Gerber, S. *et al*. Direct characterization of photoinduced lattice dynamics in BaFe_2_As_2_. *Nat. Commun.* 6:7377 doi: 10.1038/ncomms8377 (2015).
